# Bacteriocin biophysics: From protein–protein interaction models to navigators of the bacterial cell envelope

**DOI:** 10.1016/j.jbc.2026.111185

**Published:** 2026-01-22

**Authors:** Colin Kleanthous

**Affiliations:** Department of Biochemistry, University of Oxford, UK

**Keywords:** Bacteria, bacteriocin, immunity, import, cell envelope, outer membrane

## Abstract

Bacteriocins are toxins deployed by bacteria to kill their competitors. Here, I reflect on my laboratory’s work on protein bacteriocins and their immunity proteins from Gram-negative bacteria. We uncovered the structural and biophysical principles that underpin the 10-log stability range of protective immunity proteins for cytotoxic nuclease bacteriocins. We went on to elucidate how bacteriocins that kill *Escherichia coli*, *Pseudomonas aeruginosa*, and *Klebsiella pneumoniae* subvert outer membrane proteins and periplasmic energy transduction systems to drive their import. We leveraged our understanding of bacteriocin structure and function to probe the nature of the bacterial outer membrane. These studies revealed the outer membrane of *E. coli* to be an asymmetric proteolipid membrane, not an asymmetric lipid membrane as has been accepted dogma for the last 50 years. I contextualize the work with background on my career, collaborations, and academic leadership roles that influenced my development as a scientist.

My academic career of 35 years ended abruptly in September 2025 when I was forced to retire because of illness. I had been diagnosed with motor neuron disease (MND/amyotrophic lateral sclerosis) 2 years earlier. Since then, I have lost my voice but have been able to maintain my group using text-to-speech software (SpeakUnique), which did (and still does) an amazing job replicating my voice, albeit in monotone. The other physical challenges of MND however made it increasingly difficult to continue. The work surveyed in this Reflections article spans my career as a principal investigator at three UK institutions (University of East Anglia [UEA], Norwich; University of York; and University of Oxford) (Fig. 1). I summarize some of my laboratory’s discoveries in the areas of bacteriocin biophysics and cell envelope biology and highlight major changes in research direction that accompanied each move to a new institution. These changes were also the start of new collaborations, which spawned new friendships and took my work to previously unimagined destinations. Academia is fiercely competitive, so a change in research direction is a risky venture. It is no coincidence that the aphorism *illegitimi noncarborundum* often comes to mind after a failed grant or a rejected article. I have also learnt to ignore unsolicited career advice from colleagues. Better to seek out mentors without axes to grind.

**Figure 1:**
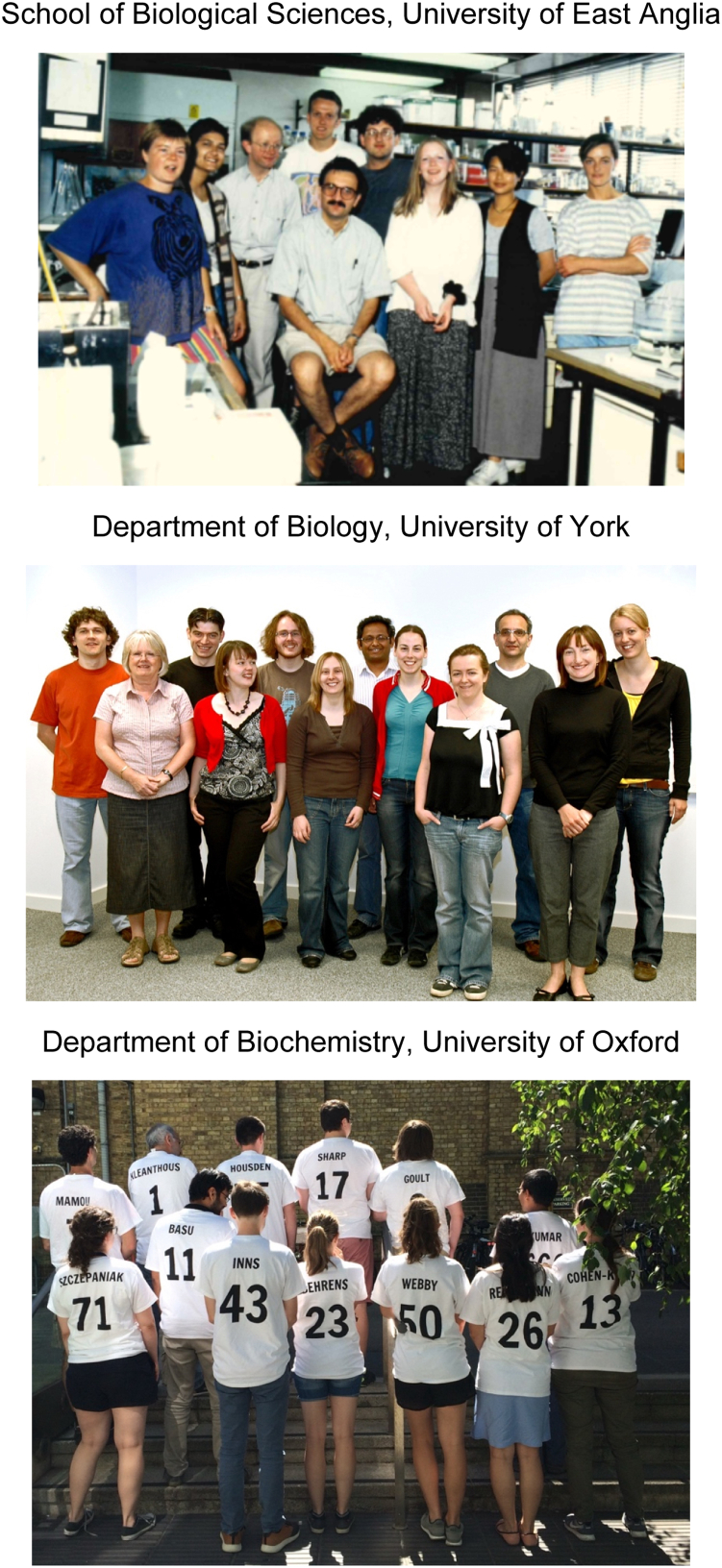
**The Kleanthous group at three UK universities.***Top*, *University of East Anglia (1994)*. *Back row, left to right,* Hortense (Tennie) Videler, Theonie (Toni) Georgio, Andrew Leech, Russell Wallis, Ansgar Pommer, Caitriona Dennis, Kit-Yi Leung, Joanna Bottomley. *Front,* Colin Kleanthous. *Middle, University of York (2008)*. *Left to right,* Konrad Zdanowski, Christina Hudson (PA), Nick Housden, Nicky Meenan, Daniel Bonsor, Katie Lilly, Amit Sharma, Anne-Marie Krachler, Nadine Kirkpatrick, Colin Kleanthous, Danielle Smith, and Luisa Stroeh (visiting Erasmus student). *Bottom, sporting lab T-shirts, University of Oxford (2018)*. *Front row, left to right,* Joanna Szczepaniak, Soumik Basu, Patrick Inns, Hannah Behrens, Melissa Webby, Nathalie Reichmann, Ruth Cohen-Khait. *Back row, left to right,* Gideon Mamou, Colin Kleanthous, Nick Housden, Connor Sharp, Jonathan Goult, and Sandip Kumar.

I was born in 1958 in North London, England, to Greek Cypriot parents, Anna and Louis. My given name is Kleanthis Kleanthous, Kleanthis being my paternal grandfather’s name. As a toddler, I had a child minder who struggled pronouncing my first name. She decided to call me Colin for reasons no one can remember. There were several famous Colins at the time, in sport, philosophy, and theater. Maybe she was a fan of one of them? Whatever the reason, it stuck, and that is how I am known except when passing through passport control. My parents emigrated to the United Kingdom in the mid-1950s. Louis was a couture tailor who fashioned garments for rich and famous women in the 1970s, 1980s, and 1990s, including royalty. Mum was a seamstress who helped dad and brought up the family. Their two aspirations for my brother Harry and me were simple: do not be tailors, get educated. They had both left school at 12 to support their families and so valued education highly. We fulfilled their aspirations, so much so that in my case, I am unable to sew even a button (and that was before MND). I went to grammar (high) school in Finchley, Margaret Thatcher’s parliamentary constituency in London, followed by the University of Leicester to study for a BSc in Chemistry with Biochemistry.

My postgraduate and postdoctoral training was in enzymology. I obtained my PhD in the laboratory of the late Bill Shaw (University of Leicester; 1985), working on the catalytic mechanism of the antibiotic resistance enzyme chloramphenicol acetyl transferase (1, 2). Toward the end of my PhD, I began thinking about forging my own academic path. For reasons best known to them, a postdoc in our laboratory working on a project unrelated to mine, took it upon themselves to advise me that I had no hope of becoming an academic. Needless to say, I ignored that advice. I went on to postdoc in the laboratories of the late Howard Schachman (University of California, Berkeley; 1985–1987) (3) and John Coggins (University of Glasgow; 1987–1990) (4, 5). This was an exciting time to be a protein chemist. Protein engineering had revolutionized life science research, and genomics and proteomics were on the way. I changed the direction of my research to prepare for this new scientific landscape. Our focus was to be protein–protein interactions and their associated biological functions, with some enzymological work where needed. My research up to that point largely centred on Gram-negative bacteria, which I continued in my group.

The first objective I set for my nascent group was an exploration of the structural and biophysical principles of protein–protein interactions. The only issue was I did not have an important (or viable) biological problem with which I could address this grand objective. Until a chance encounter with a PhD student, Russell Wallis, in the stairwell of the School of Biological Sciences at UEA. Russ asked if I could help him purify a protein that was proving problematic. The protein was Im9, which is the immunity protein for the bacteriocin colicin E9 (ColE9) (Fig. 2). Bacteriocins are antimicrobial proteins released by bacteria to kill competitors (6). Little did I know this chance encounter would be the beginning of a 35-year working relationship with bacteriocins, culminating in their weaponization to combat antibiotic-resistant bacteria through a spin-out company, Glox Therapeutics Ltd. Glox was founded by myself, Dan Walker (a former PhD student, now professor at the University of Strathclyde and Chief Scientific Officer) and CEO James Clark in 2023 (James sadly passed away in 2025). Russ was a student in the microbiology laboratory of Richard James. He subsequently came to my laboratory as one of my first postdocs, in the process switching to biochemistry and biophysics (Fig. 1). Russ had a successful academic career, becoming Professor of Molecular Immunology at the University of Leicester, working on complement activation. It was Russ who suggested a possible link between hexagonal symmetries in complement activation and the structure of the bacterial outer membrane, which I describe toward the end of this Reflections article.

**Figure 2:**
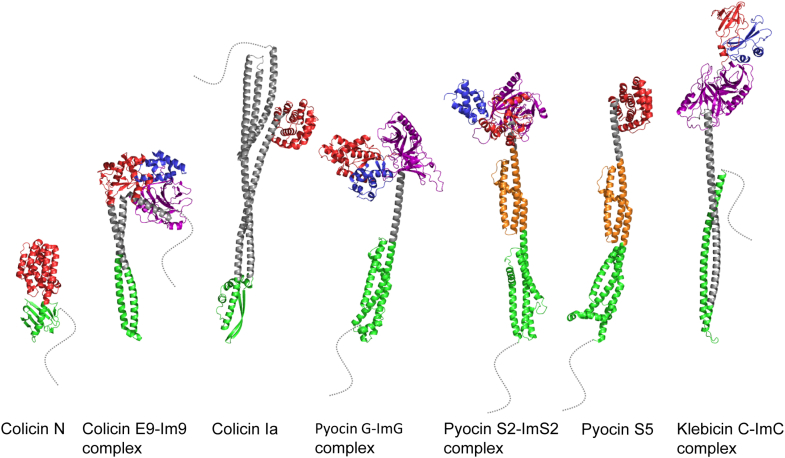
**Bacteriocin structures.** Structures of bacteriocins from different bacteria are highlighted in the Reflections article. *Escherichia coli* colicins: ColE9–Im9 complex, 62 and 10 kDa, respectively (Protein Data Bank [PDB] code: 5EW5); ColIa, 69 kDa (PDB code: 1CII); and ColN, 42 kDa (PDB code: 1A87). *Pseudomonas aeruginosa* pyocins: PyoS2–ImS2 complex, 74 and 10 kDa, respectively; PyoG–ImG complex, 69 and 10 kDa, respectively; PyoS5, 56 kDa (PDB code: 6THK). *Klebsiella pneumoniae* klebicin: KlebC–ImC complex, 66 and 10 kDa, respectively. Structures for PyoS2–ImS2, PyoG–ImG, and KlebC–ImC are derived from AlphaFold3 (141). All bacteriocins in complex with immunity proteins are nucleases; the rest are pore-forming toxins, which are not released from bacteria bound to immunity proteins. *Dotted lines* denote disordered N termini that encode either Ton or Tol import-activating signals (in ColE9, this is referred to as IUTD, Fig. 4). In the case of Tol-dependent colicins, ColN and ColE9, disordered regions also contain OmpF-binding epitopes (see text for details). In some instances, disordered regions are weakly structured but are not recognized as such by AlphaFold3; specifically, PyoS2, the N terminus of which contains a β-hairpin (see text for details and Fig. 5). Color coding of domains: *red*, cytotoxic domain; *blue*, immunity protein; *green*, receptor-binding domain; *orange*, common polysaccharide antigen-binding domain (pyocins S2 and 5 only); *purple*, inner membrane translocation (IMT) domain.

Richard had discovered the ColE9–Im9 system through an undergraduate practical class he devised (7). He was already collaborating on the system with Geoff Moore, an NMR spectroscopist in the School of Chemical Sciences. My arrival brought protein chemistry to the mix. The three of us worked on colicins and their immunity proteins for more than a decade, solving the structures of immunity proteins and the ColE9 DNase–Im9 complex, deducing the mechanism of action of colicin nucleases and dissecting specificity at the heart of colicin–immunity protein complexes. As the new millenium arrived, I yearned for a new horizon, which turned out to be the University of York. Soon after the move to York, Nick Housden joined the group and (thankfully) never left (Fig. 1). Nick has been the backbone of our bacteriocin research ever since. From the outset at York, we began delving into the translocation mechanisms of bacteriocins, especially how they tap into periplasmic energy transduction systems to catalyze their transport across the outer membrane. As part of this work, we began using fluorescently labeled bacteriocins in microscopy experiments, with the intention of following their import. It was while conducting preliminary experiments with fluorescently modified bacteriocins that we noticed the clustered organization of their receptors, which started a new area of research on supramolecular organization in the outer membrane. These discoveries coincided with my laboratory’s final move to Oxford, where we exploited the technical advances we had made to probe the nature of the bacterial outer membrane.

## Bacteriocin nucleases and their specific inhibition by immunity proteins

Bacteria frequently deploy antimicrobial systems that help shape microbial communities (8). Type VI secretion and contact-dependent inhibition rely on physical contact between cells for the toxic activity to be delivered. Bacteriocins are soluble proteins released to the environment that kill cells by receptor-mediated entry of a C-terminal cytotoxic domain (Fig. 2). Colicins are 40 to 80 kDa bacteriocins specific for *Escherichia coli* that deliver a variety of cytotoxic activities into cells (9), including pore-forming domains that depolarize the inner membrane, lipid II-hydrolyzing domains that block cell wall synthesis, and nucleases that degrade nucleic acids in the cytoplasm (DNA, rRNA, or tRNA). A similar palette of cytotoxic activities is delivered by contact-dependent inhibition and type VI secretion (10). Colicin-producing cells avoid suicide through the action of an immunity protein that neutralizes the activity of the toxin (11, 12). At the time I was setting up my laboratory, very little was known about the cytotoxic enzymatic domains of colicins or the mechanism by which immunity proteins block their activity.

**Figure 3:**
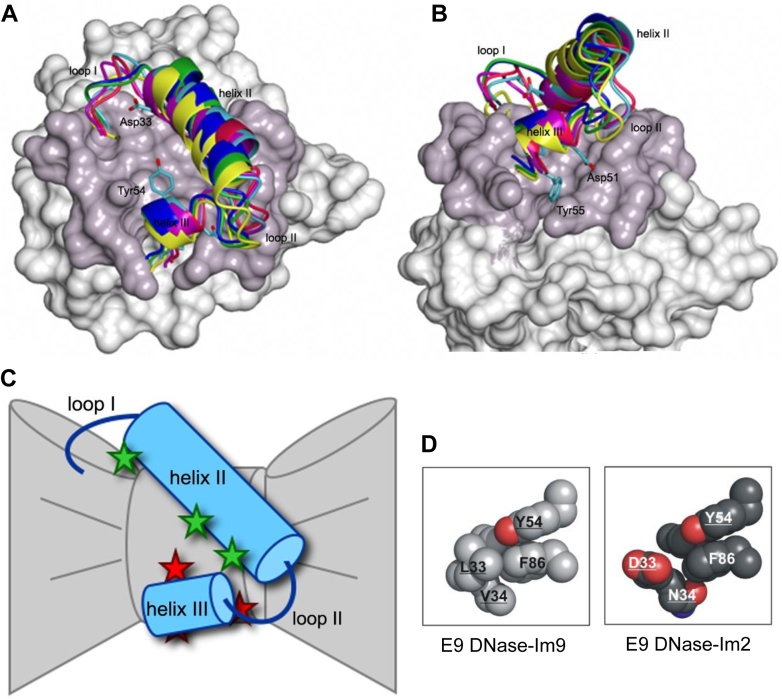
**Binding of immunity proteins to the colicin DNase exosite and chemical frustration at the heart of a noncognate complex.***A*, molecular surface representation of the colicin E2 DNase and the surface buried by its cognate immunity protein, Im2. *B*, 90° rotation. Overlayed are helices II and III, and adjoining loops, for six immunity proteins, the bound structures of which were solved by crystallography (34). Also shown are conserved helix III residues (Asp51, Tyr54, and Tyr55) that clamp the immunity protein to the base of the binding site. Asp33 in helix II is an important specificity site in Im2, which in Im9 is Leu33. The figure emphasizes the different rotameric states immunity proteins adopt when bound to colicin DNases, each presenting alternate specificity residues to the DNase from helix II and adjoining loops. *C*, cartoon depicting the bowtie nature of the immunity protein binding site on a colicin DNase. Conserved water molecules (*red stars*) form interfacial contacts between helix III and the DNase and mediate rotation of the immunity protein. Helix II forms specificity contacts, some of which are also water mediated (*green stars*). See Refs. (33, 34) for details. Panel figures from the study by Wojdyla *et al.* (34) are reprinted with permission from Elsevier. *D*, van der Waals representations of hotspot interactions at the core of the cognate ColE9 DNase–Im9 complex (*K*_*d*_ ∼10^−14^ M) and noncognate ColE9 DNase–Im2 complex (*K*_*d*_ ∼10^−7^ M). Immunity protein residues are underlined. Conserved Tyr54 from Im9 helix III is a key hotspot residue that organizes hydrophobic specificity residues involving Im9 (helix II, Leu33, and Val34) and E9 DNase (Phe86). The hydroxyl of Tyr54 is accommodated by a water-mediated hydrogen bond to the DNase backbone (not shown). In the noncognate ColE9 DNase–Im2 complex, charged and polar Im2 specificity residues (Asp33, Asn34) are forced into the same hydrophobic pocket (chemical frustration). The panel is adapted from the study by Meenan *et al.* (35).

Our early work on colicins centered on the so-called E group endonucleases (colicins E2, E7, E8, and E9) and their interactions with cognate and noncognate immunity proteins (Im2, Im7, Im8, and Im9). The active site of this group of colicin DNases is built around the well-known HNH motif, which we rechristened the ββα-Me motif after finding it was the structural core of a larger superfamily of metal-dependent nucleases (13). As well as homing endonucleases, the HNH–ββα-Me motif is found in the genome-editing enzyme CRISPR–Cas9 (14) and apoptotic endonucleases (15). We demonstrated that the ColE9 DNase binds to the minor groove of dsDNA, where it randomly hydrolyzes phosphodiester bonds using Mg^2+^ or Ca^2+^ as a cofactor (16, 17, 18, 19). The enzyme also hydrolyzes ssDNA and RNA by metal-dependent and metal-independent means, respectively. The acidic Im9 protein neutralizes these activities by binding to an exosite adjacent to the ColE9 DNase active site (20). Enzymatic activity is abolished by steric and electrostatic occlusion of nucleic acid binding. Stopped-flow fluorescence measurements revealed that Im9 associates with the DNase domain by a two-step mechanism. Rapid formation of an electrostatically driven encounter complex is followed by a slow conformational rearrangement in which the immunity protein likely rotates against the surface of the DNase (21, 22) (Fig. 3). We determined the equilibrium dissociation constant for the cognate ColE9 DNase–Im9 complex from kinetic measurements to be subfemtomolar (*K*_*d*_ ∼10^−16^ M at pH 7, 25 °C, increasing to *K*_*d*_ ∼10^−14^ in the presence of 200 mM NaCl) (23). Similar ultrahigh affinities have been reported for other nuclease–inhibitor complexes, such as the barnase–barstar complex (24).

### The basis for high affinity and high specificity in colicin DNase–immunity protein complexes

The DNase domains of colicins E2, E7, E8, and E9 share a high degree of sequence identity (∼75%) as do their corresponding immunity proteins, Im2, Im7, Im8, and Im9 (∼50%). Pre–steady-state kinetic measurements demonstrated that the association profiles and rate constants for noncognate immunity proteins binding each of the colicin DNases are similar to those of a cognate complex, but, unlike cognate immunities, dissociate rapidly. The resulting *K*_*d*_s for 16 cognate and noncognate complexes span the affinities of most protein–protein interactions in biology (*K*_*d*_s, 10^-4^–10^-14^ M) (21, 25, 26), making the system ideal to probe the basis for affinity and specificity in homologous complexes. We undertook detailed kinetic and thermodynamic studies as part of this analysis (21, 27). We uncovered a strong correlation between the affinity of colicin DNase–immunity protein complexes and the protection immunity proteins afforded to bacteria against colicin-mediated killing (25). Full immunity is conferred when *K*_*d*_ <10^−10^ M. At the time, we rationalized this affinity threshold as a simple biophysical consequence of the one-hit kinetics typically exhibited by colicins, where a single colicin successfully entering a cell results in death (25). The concentration of one molecule in an *E. coli* cell is ∼10^−9^ M. The full immunity threshold of 10^−10^ M would ensure that even one colicin molecule would be blocked from killing the cell. There are two problems with this explanation. Our recent work has shown that the first-order killing kinetics exhibited by nuclease colicins is not indicative of single-molecule killing (see below). The other problem is that cognate complexes are always >4 orders of magnitude higher affinity than the 10^-10^ M threshold. The answer is more likely to be found in how immunity specificity has evolved, which is explored below.

Immunity specificity was dissected through a combination of site-directed mutagenesis (28, 29), homolog-scanning mutagenesis (30), phage display (25), alanine scanning mutagenesis (25, 31, 32), crystallography (20, 33, 34), and computational analysis (the latter in collaboration with David Baker’s laboratory, University of Washington) (22, 34, 35) (Fig. 3, *A* and *B*). Structural and computational delineation of specificity centered on comparing different cognate complexes (ColE2 DNase–Im2, ColE9 DNase–Im9, and ColE7 DNase–Im7) (33, 34, 36) and the cognate ColE9 DNase–Im9 complex with a trapped, noncognate complex of the ColE9 DNase–Im2 complex (*K*_*d*_ ∼10^-7^ M) (35). Immunity proteins are 10 kDa, four-helix bundle proteins (37, 38) that bind at the most sequence-variable region of the 15 kDa DNase domain (11, 12). The immunity protein exosite on the DNase resembles a bowtie in structure (Fig. 3*C*). The knot of the bowtie, the narrowest part of the site, is straddled by two immunity protein helices, helices II and III, which govern the stability and specificity of the complex (34). Helix III is a conserved, one-and-a-half-turn helix, whereas helix II is long and variable in sequence and largely defines specificity (30). Immunity proteins have evolved to exploit the bowtie nature of the DNase-binding site by adopting different rotamer states. The axis of rotation for these rotamers passes through two conserved immunity protein tyrosine residues (Tyr54 and Tyr55) in helix III (33, 34), which sit above three interfacial water molecules (Fig. 3, *A*–*C*). The conserved waters not only form bridging interactions but also enable rotation of the immunity protein against the surface of the DNase. Rotation, likely the origin of the first-order process observed in all our kinetic studies, presents different immunity protein specificity sites on helix II to the DNase. Our thermodynamic studies complemented this structural and kinetic picture. We found that a dual recognition mechanism underpins immunity protein stability for colicin DNases. Helix III forms a binding energy hotspot, whereas different parts of helix II contribute to stability depending on the complex (22, 25, 31). Conserved helix III residues are accommodated at the sequence variable exosite on the DNase through hydrogen bonds to the peptide backbone, some mediated by the interfacial water molecules and van der Waals interactions (33). Extreme protein–protein interaction discrimination results from destabilizing interactions in noncognate complexes; immunity protein specificity residues in helix II adjacent to helix III are forced into chemically incompatible binding pockets (chemical frustration) (35) (Fig. 3*D*).

The late Dan Tawfik (Weizmann Institute, Israel) went further, asking how high-affinity, high-specificity protein–protein interactions evolve in colicin DNase–immunity protein complexes? His laboratory deployed *in vitro* compartmentalization, a directed evolution strategy devised with Andrew Griffiths (39), to evolve immunity protein specificity (40). They combined this approach with structural and kinetic analysis of evolved intermediates. Their study unveiled the importance of promiscuous and latent interactions as well as alternative binding modes in the evolution of high affinity and high specificity in colicin DNase–immunity protein complexes (41). Colicin DNase–immunity protein complexes have been adopted by several groups as models in computationally guided design of protein–protein interactions (42, 43, 44). Immunity proteins have also proven to be a powerful experimental system with which to investigate protein folding mechanisms, pioneered by Sheena Radford’s laboratory (Astbury Centre, University of Leeds) (45, 46).

Inspired by the diverse stabilities of colicin DNase–immunity protein complexes, I edited a book on protein–protein interactions that compared different biological systems, which together spanned a similar thermodynamic range (47). Soon after, I, along with Sachdev Sidhu, Joel Janin, Peter Hinterdorfer, Roger Goody, Walter Sebald, Gideon Schreiber, and Jacob Piehler, began making plans for a conference focused exclusively on protein–protein interactions. The first meeting of Molecular Perspectives in Protein–Protein Interactions was held in Eilat in 2005, thanks to the efforts of Gideon Schreiber. I chaired the second of these meetings in Dubrovnik in 2008. The meeting continues to be one of the conference's mainstays for the protein–protein interaction community—the eighth iteration of the meeting took place in Crete in 2025, organized by Brian Baker, Melissa Call, Mickey Kosloff, Jacob Piehler, Efstratios Stratikos, Eric Sundberg, and Beatriz Trastoy Bello.

## Bacteriocin import

The bacterial outer membrane is a rigid, load-bearing, impermeable structure that excludes several classes of antibiotics (48, 49, 50, 51). Bacteriocins are potent toxins, often active at subnanomolar concentrations, that translocate through the outer membrane. Dissecting their import mechanism requires complementary microbiological, biochemical, biophysical, and structural approaches (52). I rationalized that understanding this process in molecular detail would not only reveal how these proteins are imported but also shine a light on the cell envelope through which they pass and lay the foundations for their development as antimicrobials to combat the inexorable rise of antibiotic resistance among Gram-negative bacteria (53).

Colicins were the first bacteriocins to be identified, discovered by the Belgian microbiologist André Gratia in 1925 (54). He noticed that a strain of *E. coli* grown on a Petri dish produced a diffusible substance that killed a neighboring strain, which he called *colicine*. Bacteriocins are produced by many, if not all, bacterial species, taking the form of peptides or proteins. My laboratory focused on nuclease bacteriocins found predominantly in the Enterobacteriaceae and Pseudomonadaceae (12, 55). Protein bacteriocins (hereafter referred to as bacteriocins) are typically elongated multidomain proteins that use outer membrane proteins (OMPs), such as porins and nutrient transporters as receptors. From here, they contact one of two inner membrane, proton motive force (PMF)–linked systems, Tol and Ton, which drive entry into the cell (9). Tol (aka Tol–Pal) appears to have several physiological roles in the cell envelope of Gram-negative bacteria, but its principal role is that of stabilizing the outer membrane at division sites (56). Ton is a related system that drives the import of essential vitamins, iron–siderophore complexes, and glycans through TonB-dependent transporters (TBDTs) (57). Tol-dependent colicins, known as group A, exploit the Tol system and require the porins OmpF or OmpC to kill cells, in addition to a primary receptor. Ton-dependent colicins (group B) typically exploit a single OMP (usually a TBDT) as both receptor and translocator (58), with surface sugars sometimes used as initial docking sites (59).

As our dissection of colicin DNase–immunity protein complexes began to wind down, we switched our attention to protein–protein interactions associated with import. This change in focus coincided with my laboratory’s move to the Department of Biology at the University of York (2002), where I was appointed Professor of Biochemistry, and continued in Oxford, where I was appointed Iveagh (pronounced Ivy) Professor of Microbial Biochemistry (2012). While many of the cell envelope proteins involved in bacteriocin entry had been identified, how bacteriocins exploit them to translocate across the outer membrane was unknown (58). Unanswered questions included how they contact and exploit PMF-linked motors in the inner membrane, whether they remain bound to the outer membrane during killing and, in the case of nucleases, how they cross the inner membrane. The moves to York and then Oxford brought with them new collaborations on live-cell imaging, single-molecule tracking, mass photometry, proteomics, native mass spectrometry, cryo-EM, and molecular dynamics simulations. Access to this repertoire of biophysical and structural approaches proved instrumental (excuse the pun) in unravelling the convoluted mechanisms bacteriocins use to cross the outer membrane.

### Colicin import

**Figure 4:**
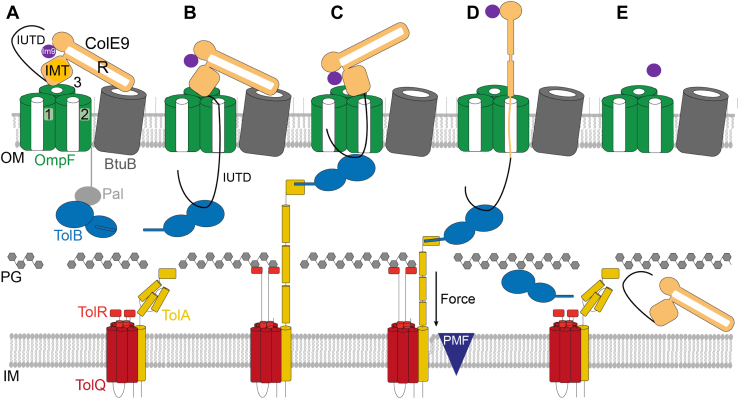
**Model for energized transport of ColE9 through the outer membrane of *Escherichia coli*.** Annotated domains of ColE9: intrinsically unstructured translocation domain (IUTD), contains two OmpF binding epitopes, either side of the TolB binding epitope (not shown). *C*, cytotoxic DNase domain; IMT, inner membrane translocation domain; R, receptor binding domain. The immunity protein Im9 (*purple*) binds and inactivates the cytotoxic DNase domain. The relative positions of the ColE9 receptor, BtuB, and translocator, OmpF, are based on the cryo-EM structure of the isolated translocon (import) complex (68). A, the ColE9 R domain binds BtuB and positions the IMT domain and its associated IUTD above a neighboring OmpF. *B* and *C*, the ColE9 IUTD enters one OmpF subunit (subunit 2) and ratchets to the periplasm from where it captures TolB from the Tol system before or after entering a neighboring OmpF subunit (subunit 1). Threading through OmpF causes ColE9 to dissociate from its receptor, BtuB, prior to energized import. The TolB binding epitope within the ColE9 IUTD allosterically modulates the conformation of TolB, releasing its N terminus to bind TolA. TolA is extended through the periplasm in an energized state by the PMF-linked TolQR inner membrane motor complex. This step involves TolR simultaneously binding the cell wall and TolA (76). *D*, retraction of TolA through the periplasm is thought to exert force on TolB, dragging unfolded ColE9 through the lumen of OmpF subunit 2 and causing Im9 to dissociate. *E*, imported ColE9 likely refolds in the periplasm prior to inner membrane translocation. Figure adapted from the study by Francis *et al.* (68), courtesy of Springer Nature. PMF, proton motive force.

The initial focus of our import studies was group A Tol-dependent bacteriocin ColE9. ColE9 is a 61 kDa multidomain DNase bacteriocin, built around a long helical coiled-coil and a ∼80 residue–disordered N terminus (60, 61) (Fig. 2). ColE3, a close homolog of ColE9 that delivers an rRNase, was the first of this family to have its complex with BtuB structurally characterized. ColE3 (and ColE9) binds with nanomolar affinity to its receptor, the vitamin B_12_ transporter BtuB, at a 45° angle relative to the plane of the membrane (62). The pore-forming colicin Ia binds its receptor Cir at a similar obtuse angle (63). Once bound to the surface, ColE9 (and ColE3) deploys its disordered N terminus like a fishing line to engage the porin OmpF in an extraordinary binding mechanism involving two linear OmpF-binding epitopes (64, 65) (Fig. 4). How BtuB and OmpF find each other in the rigid outer membrane was unknown at the time. This question has since been resolved by the discovery of supramolecular OMP organization, described below.

The two OmpF-specific epitopes bind within the pores of the same porin trimer but in opposite directions. The disordered polypeptide chain enters the lumen of one subunit and ratchets to the periplasm from where it threads back through a neighboring subunit, presenting the intervening TolB-binding epitope in the periplasm as a tethered peptide (66, 67, 68, 69). A variant of this bifurcated OmpF binding mechanism is also exhibited by the pore-forming colicin N (ColN) in its import mechanism (70). Another consequence of ColE9’s disordered N terminus threading through OmpF is the dislodging of the colicin from its receptor, BtuB, prior to energized import (68) (Fig. 4). This unusual binding mechanism was revealed through a combination of *in vivo* toxicity assays, isothermal titration calorimetry, native mass spectrometry, cryo-EM, and single-channel recording in planar lipid bilayers (the latter in collaboration with Hagan Bayley’s laboratory, Oxford). The TolB-binding epitope competes with the lipoprotein Pal for TolB to form the ColE9–BtuB–OmpF–TolB outer membrane import complex (68, 71, 72, 73, 74). TolB is a soluble periplasmic protein, and Pal is an abundant outer membrane lipoprotein that binds the cell wall unless bound to TolB. The role of the other Tol proteins, located at the inner membrane (TolA, TolQ, and TolR), is to convert the PMF into mechanical force at the outer membrane (75). This force is delivered by TolA following its association with the TolQR motor complex (76). The entire TolQRA complex is recruited to the divisome, where it captures diffusing TolB–Pal complexes in the outer membrane (77). TolB is actively dissociated from the TolB–Pal complex by energized TolA, thereby concentrating Pal at the division site and stabilizing the connection between the outer membrane and cell wall (78, 79, 80). By piggybacking TolB within the ColE9–BtuB–OmpF–TolB complex, the surface-bound colicin becomes primed for import. When force is exerted on TolB by the PMF-linked TolQRA assembly in the inner membrane, ColE9 is dragged into the cell (81) (Fig. 4).

OmpF (and its close homolog OmpC) is a trimeric porin. The lumen of an OmpF subunit (7 × 11 Å) is wide enough for the passive diffusion of small nutrients, metabolites, and antibiotics but excludes macromolecules >600 Da, including globular proteins. The lumen is however wide enough to accommodate an unfolded polypeptide chain. To test if unfolded ColE9 passes through the lumen of an OmpF subunit, rather than translocating along the external surface as had been proposed (82), we covalently attached fluorescent labels to a single cysteine residue in the C-terminal DNase domain to observe their impact on cytotoxicity (68). ColE9 can kill *E. coli* cells expressing either OmpF or OmpC, with the former being its preferred porin. Every fluorophore attached to the DNase of ColE9 abrogated OmpC-mediated killing. OmpC has a slightly narrower luminal pore (the eyelet) and a more negatively charged extracellular surface than OmpF. All the fluorophores used (AF^488^, AF^568^, and AF^647^) are negatively charged. Although OmpF-expressing *E. coli* cells could still be killed by ColE9 with fluorophores attached, the rate of first-order killing was affected significantly by the size and rigidity of the fluorophore (68). These experiments highlighted two important principles of ColE9 transport across the outer membrane. First, the colicin is transported in its entirety to the periplasm through the lumen of a porin subunit. Second, the first-order killing kinetics exhibited by ColE9 and other colicins is not indicative of one-hit kinetics, where entry of one bacteriocin molecule is sufficient to kill a cell, which had been the prevailing view. Rather, this kinetic profile is indicative of outer membrane transport being the rate-limiting step in colicin-mediated cell death. PMF-driven transport of ColE9 through OmpF also dissociates the tightly bound Im9 from the DNase domain, releasing it to the medium (Fig. 4) (83). Once transported across the outer membrane, the colicin is presumed to refold in the periplasm. There is no direct evidence of this but is inferred since denatured ColE9 readily refolds *in vitro* (84). By contrast, the lipid II–hydrolyzing bacteriocin ColM, which exploits the siderophore transporter FhuA to cross the outer membrane, requires the periplasmic chaperone FkpA for refolding (85).

The import mechanism we uncovered for ColE9 likely applies to all Tol-dependent bacteriocins, but with variations in the receptor used and Tol protein recruited for the energy-dependent step. For example, the pore-forming colicin ColN uses OmpF as both receptor and translocator and binds TolA directly for its energy-dependent transfer to the periplasm (86).

How nuclease colicins translocate from the periplasm to the cytoplasm remains unclear, but several facets of this process have been identified. The DNase and rRNase domains of colicin nucleases are basic proteins that associate with the anionic lipids of the inner membrane. This association destabilizes the domain *in vitro* and, in the case of DNases, generates non–voltage-gated ion channels in planar lipid bilayers (collaboration with Edward Lea’s laboratory, UEA) (87, 88, 89). Moreover, *in vivo* data were consistent with a direct interaction between the nuclease and the inner membrane; we found a strong correlation between the opposing charge states of the nuclease and those of inner membrane phospholipids. Thereafter, FtsH is required for the final steps of cytotoxicity (90). FtsH is an AAA^+^ ATPase/protease in the inner membrane that retrotranslocates misfolded membrane proteins to the cytosol and degrades them within a proteolytic chamber. de Zamaroczy *et al.* (91, 92) demonstrated that FtsH proteolytically releases colicin nuclease domains to the cytoplasm. They also revealed a surprising, noncatalytic role for the signal peptidase, LepB, in nuclease bacteriocin transport (93). LepB forms stable contacts with several bacteriocins (colicins and klebicins) destined for translocation across the inner membrane (94), although the role of this association is unclear. All nuclease bacteriocins have a structured domain, commonly referred to as the T-domain in bacteriocin literature. This domain, which can be positioned anywhere in the toxin except the C terminus, was thought to be involved in outer membrane transport. We demonstrated that this domain—which we renamed the inner membrane translocation (IMT) domain—is required for transfer of the nuclease to the cytoplasm (95). The same domain is also found in nuclease effectors of type VI secretion (95).

### Pyocin import

We broadened our investigations of colicin-like bacteriocin import to include the pathogens *Klebsiella pneumoniae* and *Pseudomonas aeruginosa*. *P. aeruginosa* deploys several very different types of *Pseudomonas*-specific pyocins, some related to bacteriophage tails (R- and F-type pyocins) (96). We focused on soluble, S-type pyocins that deliver similar toxic activities to *E. coli* colicins and are similarly elongated in structure (59, 60). There are no Tol-dependent S-type pyocins, presumably because of the lack of trimeric porins through which the Tol system is contacted (66). We delineated the import pathways of three TonB-dependent pyocins, the pore-forming pyocin S5 (59) and the nucleases pyocin G (97) and pyocin S2 (98) (Fig. 2). All three pyocins exploit a different TBDT for import: the pyochelin transporter FptA, the hemin transporter Hur, and the pyoverdine transporter FpvAI, respectively. As with nuclease colicins, transport of the nuclease to the cytoplasm is dependent on the IMT domain in the bacteriocin and FtsH in the inner membrane (95).

**Figure 5:**
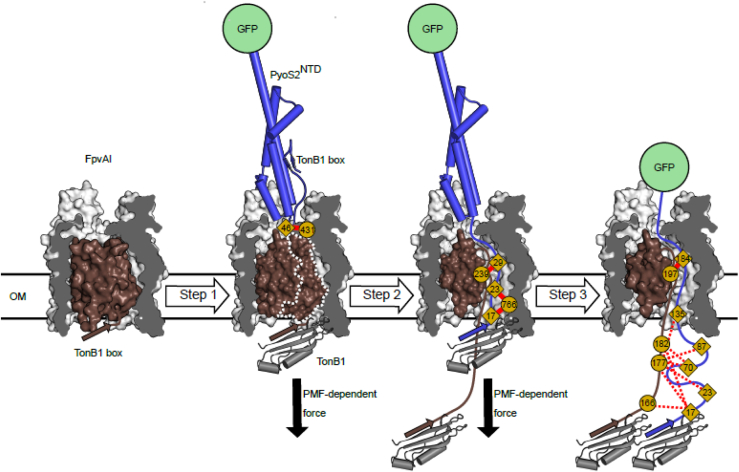
**Energized transport of PyoS2^NTD^-GFP through FpvAI in the outer membrane of *Pseudomonas aeruginosa* determined by photoactivated crosslinking.***Step 1*, binding of PyoS2^NTD^ to FpvAI mimics pyoverdine, activating the receptor for substrate transport and recruiting the C-terminal domain of TonB1 in the periplasm. *Step 2*, PMF-dependent mechanical force, applied to the receptor’s TonB1 box *via* the ExbBD–TonB1 complex, drives unfolding of the labile portion of the plug domain. Only the C-terminal domain of TonB1 is shown. The N terminus of PyoS2^NTD^ is electrostatically steered to the ∼13 Å wide cavity and ratchets to the periplasm to display its own TonB1 box (98). *Step 3*, a second TonB1 binds the PyoS2^NTD^ TonB box, and the mechanical force exerted by the ExbBD–TonB1 complex pulls the domain through the FpvAI lumen. Thereafter, further translocation is blocked by GFP. Crosslinks between PyoS2^NTD^ (*diamonds*) and FpvAI (*circles*) residues are indicated by a *red line*. Residues with multiple crosslinks are denoted by *dashed red lines*. The figure is adapted from the study by White *et al.* (98). PMF, proton motive force.

Transport across the outer membrane of *P. aeruginosa* was deconstructed for PyoS2 and its exploitation of the siderophore transporter FpvAI (98) (Fig. 5). The N-terminal domain of pyocin S2 (PyoS2^NTD^) competes with pyoverdine for FpvAI and binds the receptor with subnanomolar affinity. The 2.8 Å crystal structure of the PyoS2^NTD^–FpvAI complex showed how the pyocin, using a string of proline residues, mimics the interactions of pyoverdine, even inducing similar conformational changes in the receptor (98). We exploited this mimicry to establish a live-cell imaging import assay using PyoS2^NTD^ covalently modified at a C-terminal cysteine residue with a fluorescent green label, AlexaFluor 488. Import of PyoS2^NTD^–AF^488^ to the periplasm of *P. aeruginosa* was dependent on the PMF and the TonB1 boxes of both the receptor and the pyocin. All TBDTs have a short hydrophobic TonB box near the N terminus of the receptor, which engages TonB in the periplasm (57). *P. aeruginosa* has three TonB proteins. TonB1 engages its TBDTs. TonB is coupled to the PMF through the motor complex ExbBD, which is equivalent to the TolQR motor complex of the Tol system and a relative of the MotAB flagellum stator complex (99, 100). Energized TonB exerts a mechanical force on ligand-bound TBDTs through the TonB box, which transiently opens the receptor to allow passage of the ligand to the periplasm (101). Two technical advances enabled us to map the route of PyoS2^NTD^ through FpvAI. First, we discovered that a C-terminal GFP fusion of PyoS2^NTD^ blocked transport to the periplasm. We hypothesized that GFP was acting as a plug, trapping PyoS2^NTD^ within FpvAI in a partially translocated state. Second, the non-native, photoactivatable amino acid *para*-benzoylphenylalanine (BPA) could be incorporated into the N-terminal domain of PyoS2 without loss of pyocin cytotoxicity. Single BPA moieties were engineered into multiple sites in PyoS2^NTD^-GFP, added to *P. aeruginosa* PAO1 cells, and UV crosslinked. Crosslinked adducts were purified, and crosslink sites were mapped by LC–MS/MS (in collaboration with Shabaz Mohammed’s laboratory, Oxford) (98).

The crosslinked adducts demonstrated active transport through FpvAI. Most could only be explained by large-scale displacements (>70 Å in some instances) of PyoS2^NTD^-GFP BPA sites from their original positions in the PyoS2^NTD^–FpvAI ground state complex. Along with molecular dynamics simulations and protein engineering (102), the data revealed a multistep import process that has its origins in the force-dependent import of TBDT ligands but with nuances specific to protein import (Fig. 5). *Step 1*: Following binding of PyoS2^NTD^ to FpvAI, the TonB1 box of the receptor is engaged by TonB1, energized by the ExbBD motor complex. *Step 2*: A force-labile portion of the FpvAI plug domain that is proximal to the TonB1 box and occludes the central channel is removed by TonB1 by mechanical force. The β-hairpin housing the TonB box of PyoS2^NTD^ detaches from the body of PyoS2^NTD^ and enters the channel through electrostatic steering and ratchets to the periplasm. *Step 3*: TonB1 exerts force on the pyocin TonB box, pulling PyoS2^NTD^ into the cell. Unlike Tol-dependent bacteriocin import through OmpF, where complete unfolding is needed for the polypeptide chain to pass to the periplasm, complete unfolding of PyoS2^NTD^ is not required. PyoS2^NTD^ translocates while retaining some local secondary structure (102). The other domains of PyoS2 (IMT and nuclease domains) are presumed to be similarly transported, possibly by repeated pulls from TonB1 on the TonB1 box of the pyocin.

### Klebicin import

**Figure 6:**
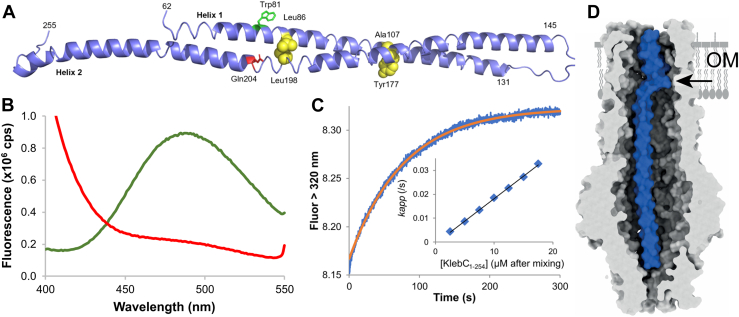
**Crystal structure of the TolC binding domain of KlebC and its association with *Klebsiella quasipneumoniae* TolC.***A*, structure of the KlebC_51–254_ helical hairpin TolC binding domain. Disulphide bonds engineered into the hairpin (Leu86, Ala107, Tyr177, and Leu198, shown as *yellow spheres*) inactivate KlebC cytotoxicity *in vivo* (105). *B*, an FRET pair was formed in KlebC_1–254_, between Trp81 (*green sticks in A*) and Gln204 (*red sticks in A*), which was mutated to Cys and modified with I-AEDANS. Fluorescence emission spectra are for 1 μM Q204C^AEDANS^ KlebC_1–254_ in the absence (*green*) and presence (*red*) of 1 μM KqTolC (λ_Ex_ = 280 nm). FRET is lost on association with TolC, suggesting the helical hairpin opens. *C*, pre-equilibrium fluorescence increase in tryptophan emission upon complex formation between 0.5 μM KqTolC and 7.5 μM KlebC_1–254_. Single exponential fit to the data to determine *k*_app_ is shown in *red*. *Inset*, dependence of *k*_app_ on KlebC_1–254_ concentration. Data are averages of duplicate experiments fitted to a straight line, the gradient of which gives the association rate constant, 1.9 ± 0.1 × 10^3^ M^−1^ s^−1^. *D*, cryo-EM structure (surface representation) showing ∼70 residues of KlebC_1–254_ bound to the lumen of the KqTolC channel (3–4 Å map resolution). KlebC_1–254_ forms a kink (denoted by the *arrow*) that enables passage through the β-barrel pore of TolC. Density for N-terminal regions of KlebC_1–254_ was not resolved beyond the iris of TolC. Figure panels were adapted from the study by Housden *et al.* (105).

The bacteriocin KlebC is a Ton-dependent rRNase toxin that targets *Klebsiella* spp. rRNase bacteriocins kill bacteria by cleaving a single phosphodiester bond within the A-site of the 70S ribosome that shuts down protein synthesis (103, 104). We identified the receptor–translocator for KlebC as the drug efflux channel TolC, using pull-down assays from *Klebsiella* extracts (105). The TolC-binding region of KlebC was narrowed down to a 27-kDa domain that included the disordered N terminus (∼50 residues) within which is the bacteriocin’s TonB box. The crystal structure of the domain revealed a 150 Å long, helical hairpin (Fig. 6*A*). Isothermal titration calorimetry demonstrated the stoichiometry of binding as one KlebC hairpin per TolC and that the interaction was high affinity (*K*_*d*_ ∼35 nM) and entropically driven. These thermodynamic parameters suggested that the KlebC hairpin unfurls to bind TolC. This interpretation was confirmed by steady-state and pre–steady-state fluorescence measurements (Fig. 6, *B* and *C*). The pre–steady-state measurements also revealed that the association rate constant for the TolC–KlebC complex is ultraslow, ∼10^3^ M^-1^s^-1^. In addition, engineered disulfide bonds across the helical hairpin abolished the cytotoxic activity of KlebC, demonstrating that opening of the helical hairpin is required for import (105).

We obtained a 3.1 Å cryo-EM structure for the KlebC N-terminal domain bound to TolC (Fig. 6*D*). The structure showed the unfurled helical hairpin of KlebC bound to TolC as a single long helix with a kink at the position of the turn (indicated by an arrow in the panel). KlebC occupied almost the entirety of the channel, except for the iris at the base of the channel where density for the N-terminal 70 amino acids of KlebC was missing. The C-terminal domains of KlebC would be positioned outside the cell, well beyond the outer membrane β-barrel of TolC. Limited proteolysis experiments were consistent with KlebC residues passing through the iris of TolC, even though they were not resolved in our structure (105). This conclusion was supported by the cryo-EM structure of another TolC-dependent bacteriocin, colicin E1, bound to *E. coli* TolC. In this instance, the unfurled helix of the helical hairpin passed directly through the iris (106). We proposed that TolC-dependent bacteriocin transport to the periplasm is a three-step process. First, the helical hairpin opens like a switch-blade knife. Second, the disordered N terminus of KlebC enters the TolC channel and ratchets to the periplasm to deliver ∼150 amino acids, ∼70 of which reside stably within the TolC channel. This step occurs spontaneously, which likely explains the ultra-slow association kinetics of the KlebC–TolC complex. Third, the TonB box of KlebC binds TonB, and the entire toxin is then pulled into the cell by PMF-coupled TonB–ExbBD assembly. The physiological role of the TolC channel is ejection of toxic substances like antibiotics and bile salts from the cell, powered by efflux pumps such as the AcrAB complex (107). We found that TolC-mediated drug efflux was partially inhibited by the KlebC helical hairpin domain, and the effectiveness of this inhibition was enhanced when the TonB box was mutated. This effect is consistent with the KlebC helical hairpin being pulled into the cell through TolC by the PMF-linked TonB–ExbBD complex. Blocking its energized import makes the hairpin a more effective inhibitor of TolC drug efflux (105).

## Supramolecular organization in the outer membrane

Gram-negative bacteria have a three-layered cell envelope comprising two membranes and an intervening peptidoglycan cell wall. In the 1970s, Nikaido (108, 109) showed that outer membrane lipids are asymmetric. Lipopolysaccharides form the outer leaflet, and phospholipids form the inner leaflet. Immersed in the outer membrane are β-barrel OMPs of varying size (8–36 β-strands) and oligomeric structure. OMPs assemble the membrane, help maintain asymmetry, form stabilizing contacts with the peptidoglycan, adhere to host cells, and mediate nutrient import, as well as being the main entry points for antibiotics (110). The standard model of the outer membrane as an asymmetric lipid bilayer, where OMPs are randomly distributed, has changed little since Nikaido.

### Binary partitioning of supramolecular OMP islands leads to OMP turnover in the outer membrane

**Figure 7:**
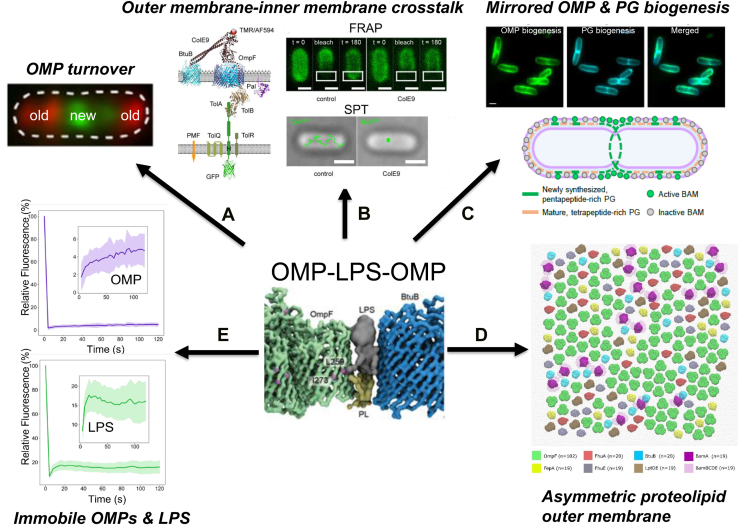
**Structure and properties of the asymmetric proteolipid membrane in *Escherichia coli* K-12.** OMP–LPS–OMP is the repeating unit of the supramolecular lattice that constitutes the outer membrane of *E. coli* and underpins much of its biology (126). The central OMP–LPS–OMP panel is adapted from the study by Webby *et al.* (126). *A*, the asymmetric proteolipid membrane is the basis for OMP turnover in growing *E. coli* by binary partitioning. New OMPs, in the form of OMP islands or clusters, are preferentially inserted at midcell, pushing old OMPs toward the poles as cells grow. Following cell division, the old poles of daughter cells contain old OMPs and the new poles new OMPs (111). The figure panel is adapted from the study by Rassam *et al.* (111), courtesy of Springer Nature. *B*, the immobility of OMPs impacts the mobility of inner membrane proteins when they are connected by a transient periplasmic protein bridge. Here, the transient protein bridge is the inner membrane protein TolA-GFP. In the absence of ColE9, TolA-GFP fluorescence recovers after photobleaching (FRAP), and single-particle tracks (SPTs) indicate random diffusion in the inner membrane. In the presence of ColE9, which binds to BtuB and OmpF in the outer membrane and TolB in the periplasm, TolA-GFP fluorescence no longer recovers after photobleaching, and SPTs are highly restricted (117). The figure panels are adapted from the study by Rassam *et al.* (117). *C*, *top*, *E. coli* OMPs and peptidoglycan were colabeled with ColB-GFP (FepA label) and the fluorescent D-amino acid, HADA, respectively, and cells were imaged by total internal reflection fluorescence microscopy. The merge panel shows how the two layers closely mirror each other. The scale bar represents 1 μm. *Bottom*, model for spatial coupling of peptidoglycan biogenesis and OMP insertion activity by the BAM (BamABCDE) complex. The figure panels are adapted from the study by Mamou *et al.* (122), courtesy of Springer Nature. Mature peptidoglycan at the old poles suppresses OMP insertion by BAM, whereas newly synthesized peptidoglycan is a poor inhibitor of BAM. *D*, coarse-grain simulation of the asymmetric proteolipid membrane containing 218 OMPs and 8093 LPS molecules (126). Every OMP, separated from every other OMP by LPS, was placed in approximate hexagonal symmetry, as dictated by the symmetrical packing of trimeric porins. See text for details. The figure panel is adapted from the study by Webby *et al.* (126). *E*, thousands of interconnected OMP–LPS–OMP repeating units, totaling ∼250,000 OMPs and >10^6^ LP molecules, render the outer membrane immobile. The panels show FRAP data for fluorescently labeled LPS and OmpF, where fluorescence does not recover after photobleaching (130). The figure panels are adapted from the study by Kumar *et al.* (130). LPS, lipopolysaccharide; OMP, outer membrane protein.

We stumbled on the spatiotemporal behaviour of OMPs while developing fluorescently labeled colicins as OMP-specific probes (111). In collaboration with Christoph Baumann’s laboratory (York), we used total internal reflection fluorescence microscopy of live *E. coli* cells to image fluorescently labeled colicins (ColE9, ColIa) bound to their OMP receptors (BtuB and Cir, respectively). BtuB and Cir were seen to cluster into islands in the outer membrane that were shunted to the poles as cells elongated (111) (Fig. 7*A*). These OMP islands also contained OMP biogenesis proteins (see below), suggesting OMPs do not move far from the biogenesis machinery that placed them there. OMP clustering and growth-dependent movement to the poles had previously been reported for the substrate-specific porin, LamB (112). We found that OMP mobility was highly confined, with measured 2D diffusion coefficients similar to those reported previously (∼0.01 μ^2^s^-1^) (113). Atomic force microscopy (AFM) measurements of porin diffusion suggest even slower mobility in the outer membrane (∼10^-7^ μ^2^s^-1^) (114). To give these values some context, inner membrane proteins typically exhibit diffusion coefficients of ∼0.1 μ^2^s^-1^. Large-scale, coarse-grained molecular dynamics simulations (in collaboration with Mark Sansom’s laboratory, Oxford) suggested that protein–protein interactions between OMPs were responsible for OMP clustering and confined diffusion (115), a point I return to below. We also discovered that new OMP biogenesis occurs primarily at the midcell of growing/dividing *E. coli*. Cell division leads to binary partitioning and retention of old OMPs at the poles of daughter cells (Fig. 7*A*). By contrast, inner membrane proteins, which typically have unrestricted mobility, are uniformly diluted among the daughter cells at each division cycle. The spatiotemporal behavior of OMPs explains how, in the absence of any means of active degradation in the outer membrane, they can be turned over simply by growth and division (111). Collectively, these studies suggest that the outer membrane has inherent structure, which is at odds with the standard model where OMPs are presumed to be distributed randomly throughout the membrane. OMPs are increasingly seen as important for the stability and integrity of the outer membrane, even though they vary in size, oligomeric structure, and abundance (51, 116). What all OMPs have in common however is that they are β-barrels. This form of tertiary structure allows disparate OMPs to be accommodated within the same supramolecular architecture of the outer membrane, as described below.

### Intermembrane crosstalk

We next asked the question, what happens to the diffusive behaviour of inner membrane proteins when they become harnessed to OMPs by transient protein bridges in the periplasm? The protein bridges in this instance were formed by the Tol system when captured by surface-bound ColE9. We found that TolA, which has unrestricted lateral mobility in the inner membrane, exhibited restricted mobility when captured *via* its association with TolB for colicin translocation (117) (Fig. 7*B*). Intermembrane crosstalk, and the inevitable subjugation of inner membrane proteins by immobile OMPs, is integral to cell envelope biology. In drug efflux by the TolC–AcrAB assembly, OMP partitioning concentrates TolC at the old pole of mother cells, making them more effective at ejecting antibiotics by efflux pumps in the inner membrane (118). When lipopolysaccharide (LPS) is secreted to the outer membrane from its inner membrane source by the Lpt system, an LPS-laden protein bridge (composed of LptA subunits) transiently connects LptDE in the outer membrane with the LPS extraction machinery LptB_2_FGC in the inner membrane. LPS modulates the kinetic stability of these bridges across the periplasm (119).

### OMP and peptidoglycan biogenesis mirror each other

Our discovery that OMP insertion into the outer membrane is not uniform but biased to the midcell of growing *E. coli* prompted an investigation into its mechanistic basis, which we conducted in collaboration with Waldemar Vollmer’s laboratory (University of Newcastle/University of Queensland). We confirmed, using 3D-structured illumination microscopy, previous reports that BamA, which catalyzes OMP insertion into the membrane in conjunction with its lipoprotein partners BamBCDE (120), is distributed throughout the *E. coli* outer membrane in the form of clusters (121, 122). OMP biogenesis however is not evenly distributed, as had been suggested previously (123). It was cell cycle dependent, implying that some BamA clusters are not viable for OMP insertion in certain parts of the cell, such as the poles. Combining *in vitro* and *in vivo* approaches, we discovered that the cell cycle dependence of OMP biogenesis is controlled by the maturation state of the cell wall (122) (Fig. 7*C*). Peptidoglycan ages as cells grow and divide. New peptidoglycan laid down at division sites, where OMP biogenesis is maximal, contains glycan chains with five amino acid stem peptides. As cells elongate, carboxypeptidases trim the stem peptides to four residues, and this form of matured peptidoglycan predominates at old cell poles (124). We found that old peptidoglycan inhibits OMP insertion activity by binding directly to BAM subunits, whereas new peptidoglycan binds poorly to BAM subunits and as a result is a poor inhibitor of OMP insertion. OMP and peptidoglycan biogenesis thereby mirror each other, leading to coordinated growth of the outer membrane and cell wall (Fig. 7*C*). In addition, we found that new peptidoglycan at division sites always preceded the emergence of new OMPs on the surface (122). We also observed coordinated growth of the cell wall and outer membrane in *K. pneumoniae* and *P. aeruginosa*, suggesting this is a fundamental principle of cell envelope biogenesis in Gram-negative bacteria. Other studies demonstrated that synchronized growth of the two layers is embedded early in cell envelope biogenesis, through the coordinated regulation of enzymes catalyzing committed steps in the biosynthesis of LPS and peptidoglycan (125).

### AFM reveals the porin network

Our fluorescence microscopy experiments pointed to some form of organization of OMPs in the outer membrane of *E. coli*, but what form this organization might take was unknown. Georgina Benn, working with Bart Hoogenboom at University College London, addressed this question using AFM of live *E. coli* cells. In their ground-breaking study, they observed a vast network of pore-like structures with pseudo-hexagonal symmetry, spread across the entire outer membrane and interspersed with patches of LPS-only regions. The pore-like structures were identified as the trimeric porins OmpF and OmpC by OmpR-modulated expression. The presence of OmpF in these networks was confirmed by ColN-mCherry labeling (ColN-mCherry does not label OmpC in live *E. coli* cells (70)). LPS-only patches, accounting for ∼10% of the surface, could split or coalesce with each other during growth, emphasizing that the outer membrane is built from distinct, phase-separated structural elements.

### OMP–LPS–OMP is the basic unit of assembly of the OMP network

How are OMPs interconnected within OMP networks? To address this question, we developed an *in vivo* crosslinking strategy to sample near-neighbor contacts of both high-abundance (OmpF) and low-abundance (BtuB and FepA) OMPs in the outer membrane of *E. coli* (126). The low-abundance OMPs are TBDTs that transport vitamin B_12_ and the siderophore ferric–-enterobactin, respectively. We introduced the photoactivatable crosslinker BPA into transmembrane regions of each OMP. We first established if OMP^BPA^ mutants were correctly folded in the membrane by assaying their susceptibility toward colicin-mediated cell death. Each of the three OMPs we targeted is exploited as a receptor by a specific colicin (ColN for OmpF, ColE9 for BtuB, and ColB for FepA). We had expected many direct OMP–OMP crosslinks based on our earlier CG simulations (115). However, of the 29 BPA sites across the three OMPs sampled, only one direct OMP–OMP protein–protein interaction was detected, between BtuB and BamA. In all other cases, where crosslinked adducts could be isolated, extensive native mass spectrometry analysis (in collaboration with Carol Robinson’s laboratory, Oxford) revealed crosslinks to phospholipids from the inner leaflet or LPS from the outer leaflet. The pattern of lipid crosslinking from the β-barrel of OmpF reflected the lipid asymmetry of the outer membrane, reaffirming Hiroshi Nikaido’s original discovery. The striking finding from these studies was that OMP crosslinks to lipids often resulted in other OMPs being copurified, particularly if the lipid was LPS. BtuB copurified with BamA (as well as being crosslinked to BamA), FepA copurified with OmpF, OmpC, FhuE, and LptD, and OmpF copurified with FepA and FhuA. These experiments suggested that OMP–LPS–OMP is the basic unit of assembly in OMP networks (Fig. 7, *central panel*), and that biogenesis OMPs (BamA, LptD) are embedded within these networks (126).

### Simulating the asymmetric proteolipid membrane

AFM, crosslinking, and proteomics data were used as restraints to build a supramolecular, 150 × 150 nm, coarse-grained model of an *E. coli* OMP island (in collaboration with Syma Khalid’s laboratory, Oxford) (126). The size of the island was based on the average size of BamA islands. Seven different OMPs were incorporated in the model, including BamA and LptD. Each OMP was present at levels consistent with published proteomics data. Of the >200 OMPs in the model, >40% were OmpF. Individual OMPs were enveloped by asymmetric lipids that were shared with neighboring OMPs and arranged in approximate hexagonal symmetry (Fig. 7*D*). The resulting near-neighbor distances for OMPs within the model matched those observed *in vivo* by AFM, which is consistent with interfacial LPS separating OMPs *in vivo*. AFM (in collaboration with Bart Hoogenboom’s laboratory, UCL) confirmed that other OMPs, specifically the TBDT FepA, reside within the porin network *in vivo*, as specified by the model (126).

These studies demonstrate that the outer membrane of *E. coli* is an asymmetric proteolipid membrane built around OMP clusters with pseudo-hexagonal symmetry. OMPs are tethered to each other by shared asymmetric lipids to create a network that covers the cell surface. Recent evidence shows that this protective lattice in *E. coli* is further fortified by the abundant peptidoglycan-binding OMP, OmpA. OmpA acts as a fence post, tethering the proteolipid lattice to the cell wall (127). As a result, OmpA integrates the compressive properties of the outer membrane with the tensile strength of the cell wall. OmpA crossbridges also generate a periplasmic osmotic pressure that helps prevent cell lysis (128). The combination of these interconnected structural elements underpins the rigidity, impermeability, and load-bearing capacity of the *E. coli* outer membrane.

While the pseudo-hexagonal symmetries evident in the outer membrane are central to its impregnability, they might also be an Achilles’ heel when it comes to surviving in mammalian hosts. IgM, the first antibody secreted in response to an infection by the innate immune system, adopts a variety of multimeric states, the most active of which in complement activation is hexameric (129). Although an intriguing hypothesis, no evidence, to my knowledge, exists in support of hexagonal symmetry in the bacterial outer membrane and susceptibility toward complement.

### OMP and LPS insertion dynamics are fundamentally different

The three principal components of the bacterial outer membrane, OMPs, phospholipids, and LPS, come together to form the asymmetric proteolipid membrane in Gram-negative bacteria. Phospholipids can diffuse laterally in the inner leaflet of the membrane, whereas OMPs and LPS cannot. The immobility of OMPs and LPS is key to the integrity of the supramolecular network (126). How might such a static structure be assembled in growing bacteria? As a first step toward understanding how the proteolipid network is assembled, we investigated the surface distribution and insertion dynamics of OmpF, one of the major OMP constituents of the network, and LPS. OmpF was labeled with fluorescent ColN, and LPS was labeled using click chemistry.

Diffraction-limited fluorescence microscopy confirmed both macromolecules to be in the cell periphery and immobile (by fluorescence recovery after photobleaching experiments) (Fig. 7*E*). Super-resolution fluorescence microscopy demonstrated both OmpF (visualized by 2D-PALM–total internal reflection fluorescence microscopy following labeling with ColN fused to photoactivated mCherry) and LPS (visualized by 3D-dSTORM of AF^647^-labeled LPS) are deposited in the membrane as clusters by clusters of their biogenesis proteins, BamA and LptD, respectively (130). Notwithstanding its abundance, OmpF exhibited the same cell-cycle insertion dynamics as observed previously for low-abundance OMPs. Microfluidics (Mother machine) experiments (in collaboration with Stephan Uphoff’s laboratory, Oxford) showed that fluorescently labeled LPS was diluted ∼50% at each division cycle, with only minor accumulation of old LPS at the poles. Since LPS is immobile, this result implies that new LPS dilutes old LPS by constantly being incorporated across the outer membrane. Hence, the spatiotemporal insertion dynamics of LPS and OMPs are completely different (130).

LPS secretion by the Lpt system is a complex process. ATP hydrolysis by the LptB_2_FG complex drives LPS extraction from the inner membrane, delivery to periplasmic LptC, and transport along the LptA protein bridge, and ultimately insertion into the outer membrane by LptD, in association with the lipoprotein LptE. The process (likened to a PEZ candy dispenser (131)) relies on stacked LPS molecules on the bridge pushing LPS molecules ahead of them till they emerge from LptD in the outer membrane. We speculated that the same pushing principle might also overcome local immobility in the outer membrane. In this scenario, LPS molecules emerging from LptD would push other LPS molecules already in the membrane and possibly even OMPs.

## Concluding remarks and further questions

Our exploration of bacteriocin biophysics answered some questions, unearthed many new ones, and reminded us of well-known questions yet to be answered. We established the basis for high affinity and high specificity in colicin DNase–immunity protein complexes. We also established how bacteriocins navigate their way across the outer membrane by exploiting PMF-linked Tol or Ton assemblies, jettisoning the tightly bound immunity protein along the way. However, the actual process of energized import remains a mystery. Several structural studies have defined how the force transducer proteins, TolA and TonB, associate with their motor complexes, TolQR and ExbBD, respectively, in the inner membrane, and the likely path protons take through these PMF-driven assemblies (76, 100). It has also been established that these are rotary motors that contact the peptidoglycan (76, 81, 132, 133). But how mechanical force is generated at the outer membrane by their force transducer proteins is unclear. Even more perplexing is how these forces are harnessed to transport polypeptides significantly longer than those associated with endogenous function. TonB, for example, dislodges ∼50 to 60 amino acids from the plug domain of TBDTs to import nutrients, whereas bacteriocins transported through the same TBDTs are more than 10 times this length. In both cases, a single TonB box located near the N terminus of the polypeptide chain suffices.

For bacteriocins that generate pores in the inner membrane or hydrolyze the lipid II peptidoglycan precursor, their journey through the cell envelope ends in the periplasm. The journey of nuclease bacteriocins however continues, taking them across the inner membrane. This process is poorly understood, other than it requires the IMT domain (95), involves the nuclease associating with the inner membrane (87, 88, 89), sometimes in association with LepB (94), and is proteolytically released to the cytoplasm by FtsH (90, 92). How the bacteriocin escapes complete degradation by FtsH is not known. Similarly, what role the IMT domain plays in the transport step is unknown. A notable feature of the inner membrane transport step is that it is faster than transport across the outer membrane, which is rate limiting for nuclease bacteriocin–mediated cell death.

The outer membrane of a Gram-negative bacterium is often represented in reviews and textbooks as an asymmetric lipid bilayer, sometimes with no OMPs at all. This depiction is incorrect. There is an abundance of biophysical data for the model organism *E. coli* K-12 demonstrating its outer membrane is predominantly an asymmetric proteolipid membrane. OMPs, enveloped by asymmetric lipids, form a lattice with pseudo-hexagonal symmetry that covers much of the cell surface, with patches of LPS-only regions breaking up the lattice (114, 126). Purified trimeric porins, which are some of the major OMPs in *E. coli*, form highly ordered 2D crystalline arrays with hexagonal symmetry in the presence of lipids (134). The fluidity of the lipid membrane enables the free energy of the system to be minimized, maximizing the surface of contact between the trimers. The hexagon-like arrangements of porins observed by AFM in the outer membrane of *E. coli* are decidedly imperfect (114), yet their presence in a membrane that is not fluid is informative. It suggests that a lateral mixing force overcomes local outer membrane immobility, enabling OMPs within reach of this flow to approach hexagonal symmetry. We suggest that this lateral mixing force results from LPS being injected into the outer membrane from different directions by multiple LptDs. See Figure 7*D* for a hypothetical arrangement of multiple LptDs in a pseudo-hexagonal arrangement with other OMPs. From our limited sampling of inter-OMP associations by BPA crosslinking, TBDTs, BamA, and LptD all share lipids with OmpF–OmpC (our strategy negated capturing OmpA) (126). We assume that the entire outer membrane proteome is accommodated within the porin lattice, which likely also contributes to imperfections in outer membrane symmetry. How does the absence of trimeric porins, for example, in *P. aeruginosa*, affect the symmetry and biophysical properties of the membrane? Does symmetry in the bacterial outer membrane have any influence on susceptibility to the innate immune system?

The *E. coli* outer membrane is estimated to contain ∼2 × 10^6^ LPS molecules, equating to ∼70,000 LPS/min being incorporated during exponential growth (135). LPS molecules are delivered by ∼125 LptA bridges to ∼1000 LptDs (in defined media) in the outer membrane (119, 136). Each LptD is only transiently connected to a bridge (5–10 s), during which time ∼10 LPS/s are delivered, driven by the hydrolysis of one ATP per LPS (119). Since LptD (which is immobile) outnumbers LptA bridges ∼10-fold, transient dynamics ensures the inner membrane components, which are mobile, can service them all. This transient delivery system is the basis for the constant injection of LPS across the entire outer membrane. Proteomics estimates that the total number of OMPs being enveloped by this LPS in the *E. coli* outer membrane, in minimal media, is ∼250,000 (136). Our computed model of the *E. coli* asymmetric proteolipid membrane suggests that LPS needs to outnumber OMPs by ∼40-fold (Fig. 7*D*), yet the number of LPS molecules, by current estimates, only outnumber OMPs by eightfold. This discrepancy is likely to increase further when LPS patches are included in calculations. Differences such as these will need to be reconciled if assembly of the asymmetric proteolipid membrane in Gram-negative bacteria is to be fully understood.

How do OMP islands observed by fluorescence microscopy relate to the expansive proteolipid network observed by AFM? Using fluorescence photobleaching, we estimated the average number of TBDT molecules in colicin-labeled islands (FepA and BtuB) to be 20 to 30 (126). Hence, the islands observed by light microscopy are tiny by comparison to the proteolipid network seen by AFM. These clusters or islands are inserted by BamA clusters, which are responsible for the clustered insertion of all OMPs as well as that of LPS since LptD is a BamA substrate (137). How many BamAs and LptDs are present in their clusters? How are BamA clusters formed (the chicken-and-egg problem), and what governs their distribution? The material inserted by BamA and LptD clusters presumably overlaps, ensuring the proteolipid network covers the surface. Are LPS patches the consequence of these clusters not overlapping? Are LPS patches seen in the outer membranes of other Gram-negative bacteria? Do they have a physiological role, for example, as sites of OMP insertion or as disruptors of the rigid proteolipid lattice to ease deformation of the outer membrane during cell division?

During my early work on bacteriocins in the 1990s, I was advised by a senior bacterial toxinologist to give up this line of research because unlike toxins like shiga or botulinum, it was not fundable as bacteriocins do not kill people. I ignored this advice because I found these molecules and what they do fascinating. Luckily for me, funders agreed, some of the time. As a result, we deduced how bacteriocins cross the bacterial outer membrane. Meanwhile, Dan Walker’s laboratory in Glasgow demonstrated that bacteriocins selectively kill pathogens in animal infection models (138, 139). These combined advances were the foundations for Glox Therapeutics Ltd (https://gloxtherapeutics.com), a precision antibiotics spin-out company targeting antimicrobial resistance in Gram-negative bacteria.

In some of his early pictorial representations of the outer membrane, Hiroshi Nikaido recognized that OMPs would be densely packed in the asymmetric lipid bilayer he had discovered (140). The finding that OMPs are integral to outer membrane structure and stability is therefore an old idea reborn. In the intervening 50 years since the discovery of lipid asymmetry, we have learnt some of the basic ground rules for the biogenesis of this complex structure in living bacteria. In the not-too-distant future, it may be possible to exploit these ground rules for the creation of new classes of antibiotics that explicitly target assembly of the asymmetric proteolipid outer membrane.

### External roles—The Wellcome Trust and Biochemical Society

Most academics support the community of their discipline by serving on funding and society panels. Of the numerous national and international organizations I have had the pleasure of working with, the two that stand out are The Wellcome Trust and The Biochemical Society. I became chairman of the Basic Science Interview Committee (BSIC) at the Wellcome Trust (2010–2012) and chairman of the Biochemical Society (2011–2013) around the time I was contemplating a move to Oxford. These roles were preceded by 3 years as a member of the BSIC and 3 years as the vice-chairman of the Biochemical Society. I found these roles immensely rewarding, not only because they helped support the life science community but also just because it was enjoyable working with the other academics and professional staff within these organizations, many of whom became friends. These gilt-edged experiences also taught me how to (and how not to) chair committees, a transferable skill strangely absent from the plethora of training programs universities are fond of making their academics complete. BSIC was the fellowship panel at the Trust (since superseded by other panel structures) run by Candy Hassall, a name that will be familiar to anyone of my dotage who applied to Wellcome Trust for funding. Gin and tonics after BSIC panel meetings, followed by dining in London’s West End, live long in the memory. The Biochemical Society numbered ∼5000 members when I became chairman. I was supported in this role by the society’s CEO, Chris Kirk, and, following Chris’s retirement, Kate Baillie. I count myself immensely fortunate that my chairmanship coincided with Chris and Kate’s time at the Society, both of whom were exceptional CEOs. The first year of my chairmanship coincided with the Society’s Centenary. (https://portlandpress.com/biochemist/article/32/6/46/803/Celebrating-100-years-of-The-Biochemical-Society). We had organized events throughout the year to celebrate the centenary, culminating in a pre-Christmas conference at the Royal Society, which also hosted the conference dinner. Surprise after-dinner entertainment had been arranged as the final event of the centenary, which almost turned into a disaster. As I gave out the last of the awards and prizes, one of the waiters took the microphone and asked the ∼100 attendees to sing happy birthday to one of the waitresses. The bewildered audience was about to break into a song when one of the distinguished scientists at the meeting (who will remain nameless) took exception to the waiter’s rude and presumptuous behavior. He made a grab for the microphone to stop the disruption, as he saw it. A minor scuffle broke out for ownership of the microphone. Calm was eventually restored. Happy birthday was finally sung, and the waiters and waitresses, who had staffed the cloakroom and served dinner all evening, were able to deliver the last event of the centenary, a wonderful medley of opera arias. It was a memorable night in a memorable year.

## Conflict of interest

C. K. owns equity in the antibiotic development company Glox Therapeutics Ltd. The author declares no conflicts of interest with the contents of this article.
